# Assessment of knowledge and practice of dentists towards Coronavirus Disease (COVID-19): a cross-sectional survey from Lebanon

**DOI:** 10.1186/s12903-020-01273-6

**Published:** 2020-10-13

**Authors:** Zeina Nasser, Youssef Fares, Rama Daoud, Linda Abou-Abbas

**Affiliations:** 1 Faculty of Medical Sciences, Neuroscience Research Center, Lebanese University, Hadath, Lebanon; 2 Faculty of Sciences, Lebanese University, Hadath, Lebanon

**Keywords:** Knowledge, Practice, Dentist, Coronavirus, COVID-19, Lebanon

## Abstract

**Background:**

Coronavirus Disease (COVID-19) epidemic is a public health emergency of international concern. Dentists are exposed to the enormous risk of COVID-19 infection during this epidemic. This study aims to assess the knowledge and practice of dentists toward the COVID-19 epidemic in Lebanon.

**Methods:**

We conducted an online survey using the snowball-sampling technique. Information on socio-demographic data, knowledge, practice, and additional information required concerning COVID-19 were collected.

**Results:**

Our results showed that the majority of the Lebanese dentists had good knowledge (91.3%), and nearly half of the respondents had a good practice (58.7%) regarding COVID-19. The most common information source was the World Health Organization (73.7%). Multiple linear regression showed that specialist dentists who completed training on COVID-19 with a high level of knowledge had better practice.

**Conclusions:**

Lebanese dentists revealed good knowledge regarding COVID-19. However, dentists had limited comprehension of the extra precautionary measures that protect the dental staff and patients from this virus. Our findings have important implications for the development of strategies suitable for improving the level of practice among dentists and enhance prevention programs.

## Background

Coronavirus Disease (COVID-19) outbreak was first reported in Wuhan, China, in December 2019 [1]. The novel virus Severe Acute Respiratory Syndrome Coronavirus 2 (SARS-CoV2) has rapidly spread from Wuhan to most of Hubei province and subsequently to the rest of the countries [2]. On January 30, 2020, the World Health Organization (WHO) declared that Coronavirus outbreaks have constituted a global health emergency of international concern [3].

Coronavirus is primarily transmitted during close contact from human to human through respiratory droplets. It is transmitted directly from person to person when a COVID-19 case coughs or exhales producing droplets that reach the nose, mouth, or eyes of another person. Other people become infected with the virus by touching fomites, then touching their face [4]. The risk of cross-infection between dentists and patients constitute major concerns in dental clinics. Treatment procedures which involve the use of rotary dental and surgical instruments such as handpieces or ultrasonic scalers and air–water syringes and others [5] are a direct route for virus spread [6]. Therefore, the standards protective measures in daily dental work are not effective enough to prevent the potential spread of coronavirus especially when a large number of droplets and aerosols are emitted from asymptomatic cases. Thus, strict infection control protocols in dental settings are urgently needed in countries affected by COVID-19. To ensure a safe working environment and to prevent transmission of COVID-19 in dental practice, the Centers of Disease Control and Prevention (CDC) [7] and the WHO developed guidelines, which mainly included standard precaution to control the spread of COVID-19.

Lebanon is taking part in the global fight against the COVID-19 pandemic. Its first case was confirmed on February 21, 2020. By May, 5th 2020, the virus has affected 741 cases [8]. Although there were no reported cases of coronavirus transmission in a dental setting in Lebanon, dentists must be constantly aware of threats that challenge their current practice. Therefore, an assessment of their knowledge is important to identify existing gaps in infection control practice.

Our aim in this study is to assess the knowledge and practice of dentists toward COVID-19 epidemic in Lebanon and to provide effective recommendations for dental practitioners.

## Methods

### Study design and population

We conducted an online cross-sectional survey using the snowball-sampling technique during April 2020. As the Lebanese Government recommended the public to minimize face-to-face interaction and isolate themselves at home, potential respondents were electronically invited to participate. A link was sent to the participants including a brief introduction on the background, the objective of study, voluntary nature of participation, declarations of confidentiality and anonymity, and instructions for filling in the questionnaire. We invited all Lebanese dentists, working in private clinics, medical centers, or hospitals to participate in the study. Any paramedical staff and dental students were not included in this survey.

### Sample size calculation

The sample size was calculated using the online Raosoft sample size calculator designed specifically for population surveys. Assuming 5000 dentists are actively practicing, the required calculated sample size was 357 with a confidence level of 95% and a 5% margin of error. The response acceptance was closed (April 30, 2020) when the required sample size was achieved.

### Data collection

The questionnaire of the survey was developed by the authors (Additional file 1) after reviewing the relevant published literature [9] and the most recent available information on COVID-19 from the international guidelines [7, 10, 11]. The questionnaire was initially designed in the English language to cover important aspects of the knowledge and practice of dentists toward COVID-19. An independent committee consisting of five experts with an expertise in implementing infection control measures, dental practice, and epidemiology reviewed in-depth the first draft of the questionnaire. They assessed the relevance of the items regarding dentists’ knowledge and practice. A consensus was reached, and a preliminary final version of the questionnaire was developed. Then, the items were translated and adapted to the Arabic language by translators. A structured questionnaire consisted of four sections:

1. Baseline characteristics included were age, gender, marital status, designation and years of practice, complete training on COVID-19.
2. The knowledge section consisted of 14 questions (true, false and don’t know) and was aimed to assess and evaluate the general knowledge of dentists about the route of transmission, signs and symptoms, risk factors, treatment, precautionary measures, and prevention. A correct answer was assigned 1 point and an incorrect/don’t know answer was assigned 0 points. The total knowledge score was 14 ranged from 0 to 14, with a higher score indicating a better knowledge. Dentists with knowledge score above 60% were regarded as having good knowledge, while those who scored below 60% were considered having poor knowledge based on Bloom’s cut off point [12]. Respondents were also asked to indicate their main source of information regarding COVID-19.
3. The practice section included 11 questions to evaluate the actual compliance and application of various preventive measures. We collected information about their practice in the clinic after announcing the first positive corona case in Lebanon. Practice scale is scored 2, 1, and 0 for “always”, “occasional” and “never” respectively. A score of 1 was given for choosing the answer reflecting good practice and 0 was given for choosing the answer reflecting poor practice. Participants with scores > 80% were classified as having acceptable preventive practice, while those with scores < 80% were considered having an unacceptable preventive practice based on Bloom’s cut off point [12].
4. The last section included 4 questions to assess the fear among dentists regarding COVID-19 and 1 question to evaluate their perception toward the actions implemented by the Lebanese order of dentists to fight the disease.

### Procedure

An Arabic questionnaire was designed on google forms and a link was shared with Lebanese dentists. The survey was pilot tested in a sample of 10 dentists to check the clarity of all items. Dentists did not report any problems in understanding the questionnaire. On average, the survey was completed within approximately 7 min. The data of the pilot study was removed from the final analysis.

### Statistical analysis

Descriptive statistics were reported using means and standard deviations (SD) for continuous variables and frequency with percentages for categorical variables. Multivariate linear regression was used to identify factors associated with practice score as a dependent variable. Unstandardized regression coefficients (β) and their 95% confidence intervals (CIs) were reported. Statistical analysis was carried out using the statistical software SPSS (Statistical Package for Social Sciences), version 22.0. All tests were two-tailed, with a significance level of P value < 0.05.

## Results

### Characteristics of the study participants

The baseline characteristics of the participants are shown in Table 1. The study included 358 dentists working in private clinics, medical centers, or hospitals with a mean experience of 10.3 ± 8.1 years. Their mean age was 34.92 ± 9.2 years ranging from 23 to 65 years. There was (54.2%) male respondents. By designation, more than half of the dentists (61.2%) were specialists. The majority of the dentists reported that they didn’t get any training on COVID-19 (95.5%) and that special dental clinics should be allocated to treat COVID-19 (89.7%).

**Table 1:** Demographic characteristics of the study participants (N = 358)

Variable		Min–max	Frequency	Percentage
Age (mean ± SD) years	(34.92 ± 9.2)	23–65		
Clinical experience (mean ± SD) years	(10.3 ± 8.1)	1–40		
Gender				
Male			194	54.2
Female			164	45.8
Designation				
General dentist practitioner			139	38.8
Specialist			219	61.2
Completing training on COVID-19				
No			342	95.5
Yes			16	4.5
Treating COVID-19 patients in special dental clinics				
No			37	10.3
Yes			321	89.7

*SD* standard deviation

### Dentists’ knowledge towards coronavirus

Table 2 describes the dentists’ answers regarding COVID-19 knowledge. Out of the 358 dentists, the majority (91.3%) had good knowledge. Poor knowledge was more obvious in questions related to the incubation period of coronavirus, the transmission of the disease, the actions in dealing with suspected, probable and confirmed cases and precautionary measures by dentists in which the wrong responses rate were 38.0%, 43.0%, 35.2%, and 38.5% respectively. The mean total knowledge score was 10.56 ± 1.56.

**Table 2:** Dentists’ knowledge toward COVID-19 (N = 358)

Items	Response		
	Correct n (%)	Wrong n (%)	Don’t know n (%)
K1. The incubation period of Coronavirus is 1–21 days	136 (38.0)	214 (59.8)	8 (2.2)
K2. The main symptoms of Corona are fever > 38 °C, cough, sore throat, runny nose and shortness of breath	336 (93.9)	22 (6.1)	0 (0)
K3. Corona virus does not infect children	316 (88.3)	23 (6.4)	19 (5.3)
K4. Covid-19 can be prevented by administration of a vaccine	341 (95.3)	4 (1.1)	13 (3.6)
K5. Covid-19 is transmitted through direct contact with respiratory tract secretions	308 (86.0)	47 (13.1)	3 (0.8)
K6. Covid-19 can persist on surfaces for a few hours or up to several days	326 (91.1)	26 (7.3)	6 (1.7)
K7. Covid-19 can be transmitted through eating undercooked meat/chicken	154 (43.0)	133 (37.2)	71 (19.8)
K8. The disease cannot be transmitted from asymptomatic patients	319 (88.3)	33 (9.2)	9 (2.5)
K9. The use of Personal protective equipment (including masks, gloves, gowns and goggles or face shields) is recommended to protect skin and mucosa from (potentially) infected blood or secretions	345 (96.4)	5 (1.4)	8 (2.2)
K10. Hand hygiene has been considered the most critical measure for reducing the risk of transmitting of Coronavirus to patients	354 (98.9)	3 (0.8)	1 (0.3)
K11. All surfaces contaminated by the patients with Covid-19 infection should be cleaned with diluted (5%) bleaching solution	126 (35.2)	182 (50.8)	50 (14.0)
K12. Dentists should take strict personal protection measures and avoid or minimize operations that can produce droplets or aerosols	351 (98.0)	4 (1.1)	3 (0.8)
K13. PPE donning sequence: (1) gown (2) mask (3) gloves	232 (64.8)	83 (23.2)	43 (12.0)
K14. PPE removal sequence: (1) gloves (2) gown (3) mask	138 (38.5)	169 (47.2)	51 (14.2)

n frequency, % percentage

### Source of knowledge

Table 3 summarizes the sources of information utilized by dentists to seek information regarding COVID-19. The majority of participants reported the World Health Organization (73.7%) as the main source of knowledge and a considerable percent depend on the Ministry of Public Health (MOPH) (52.8%) and Television (TV) (44.7%) respectively. The least source was CDC (19%).

**Table 3:** Source of information regarding COVID-19

Source of information^a^	n (%)
WHO	264 (73.7)
MOPH	189 (52.8)
TV	160 (44.7)
Social media	154 (43)
Lebanese order of dentists	99 (27.7)
CDC	68 (19)
Others	111 (31)

n frequency, % percentage

*WHO* World Health Organization, *MOPH* Ministry of Public Health, *TV* television, *CDC* Centers for disease control and prevention

^a^Multiple responses

### Dentists’ practice towards Coronavirus after the announcement of the first case of COVID-19

Table 4 shows the responses of the participants regarding the various preventive practices after the announcement of the first case of COVID-19 in Lebanon. The overall mean practice score was 8.32 ± 2.02 and the Cronbach alpha for its internal consistency was 0.8. Nearly half of the studied sample (58.7%) reported good practice and 41.3% reported poor practice toward COVID-19. Most of the dentists practiced appropriate protective measures in their clinics; including changing gloves after each patient (100%), cleaning hands by using alcohol-based hand rub or soap and water (98.9%), and washing hands before and after patient treatment (98.9%). A lower percentage of good practice was observed among dentists in providing patients with alcoholic disinfectants and masks in the waiting rooms (66.8%), wearing the personal protective equipment by the assistant (65.9%), and disinfecting all surfaces, chairs and doors of the waiting room every 2 h with Chloride solution or any type of sterilizer (62.8%).

**Table 4:** Dentists’ correct responses regarding practice after the announcement of the first case of COVID-19 (N = 358)

Items	Response	
	n	%
P1. I clean my hands by using alcohol-based hand rub or soap and water	354	98.9
P2. I Clean and disinfect environmental surfaces	344	96.1
P3. I Wear the personal protective equipment such as dental goggle, mask, gloves, face shield, head cover and feet cover (dentist)	278	77.7
P4. I wear the personal protective equipment such as dental goggle, mask, gloves, face shield head cove and feet cover (assistant and team)	236	65.9
P5. I wash my hands before and after patient treatment	354	98.9
P6. Change gloves after each patient	358	100
P7. I Perform hand hygiene Before putting on gloves and again immediately after removing gloves	349	97.5
P8. I Avoid busy clinic and I give separate appointments	311	86.9
P9.I provide patients with alcoholic disinfectants and masks in the waiting rooms	239	66.8
P10. I Disinfect all surfaces, chairs, and doors of the waiting room every 2 h with Chlore solution or any type of sterilizer	225	62.8
P11. I Disinfect the patient’s chair and light between the patient and the other	287	80.2

n frequency, % percentage

### Fear of dentists towards COVID-19

**Fig. 1:**
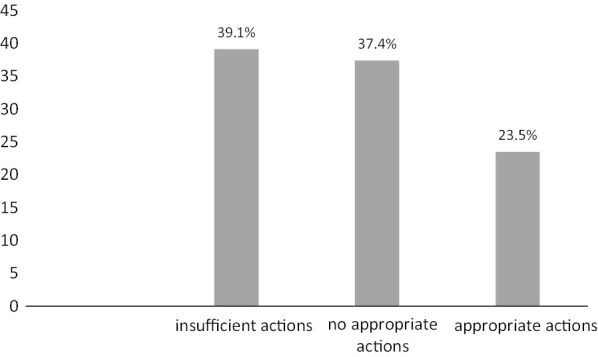
Dentists’ Perceptions towards policies/actions implemented by the order of dentists in fighting COVID-19

Table 5 is a description of the fear of dental care professionals toward COVID-19. More than 80% of the dentists were afraid to treat a patient suspected or confirmed infected with COVID-19 and were afraid of getting infected with COVID-19 from a colleague. The vast majority of the participants (95.8%) were afraid of the impact of the COVID-19 crisis on dentists’ livelihood. Figure 1 shows that 37.4% of the dentists declared that no actions were taken by the order of dentists regarding COVID-19, 39.1% reported that actions were insufficient while only 23.5% reported that the actions were acceptable/appropriate to combat COVID-19.

**Table5:** Fear of dentists toward COVID-19

Items	Response	
	Yes n (%)	No n (%)
Are you afraid to treat a suspected or confirmed patient with COVID-19 in your clinic?	309 (86.3)	49 (13.7)
Are you afraid of getting infected with COVID-19 from a colleague?	293 (81.8)	65 (18.2)
Did your assistant express his/her desire to stop work due to fear of infection with the Coronavirus	330 (92.2)	28 (7.8)
Are you afraid of the impact of COVID-19 crisis on dentists’ livelihood	343 (95.8)	15 (4.2)

n frequency, % percentage

### The relation between characteristics of the participants and practice score

The bivariate analysis showed that specialty and completing a COVID-19 training were highly associated with the practice score (P value < 0.0001). We also found that the fear of getting infected while treating a suspected or infected patient is associated with a practice score (P value < 0.0001). Moreover, the Pearson correlation test revealed a highly statistically significant positive linear correlation between knowledge and practice scores (r = 0.3, P value < 0.0001). The practice score didn’t differ statistically with age, gender, marital status, and clinical experience (P value > 0.05) (Table 6).

**Table 6:** Association between characteristics of the participants and practice score

	Practice	P value
Gender ^a^		0.885
Male	8.32 ± 2.0	
Female	8.29 ± 2.0	
Marital status^a^		0.712
Married	8.34 ± 2.0	
Others	8.26 ± 2.0	
Designation^a^		< 0.0001*
General practitioner	8.7 ± 1.7	
Specialist	8.0 ± 2.1	
Completing a COVID-19 training		< 0.0001*
No	8.25 ± 2.0	
Yes	9.56 ± 0.62	
Afraid to treat a suspected or confirmed patient infected with COVID-19^a^		< 0.0001*
No	9.01 ± 1.35	
Yes	8.16 ± 2.1	
Knowledge score^b^	0.3	< 0.0001*
Age^b^	0.8	0.12
Clinical experience^b^	0.02	0.63

^*^P value < 0.05 is considered significant

^a^Student *t* test

^b^Pearson correlation

### Factors associated with practice score regarding COVID-19

Multiple linear regression analysis was presented in Table 7. Our results showed that high level of knowledge, trained and specialist dentist had better preventive practice regarding COVID-19 (beta = 0.319, 95% CI (0.192–0.445); P value < 0.0001, beta = 1.234, 95% CI (1.238–2.185); P value = 0.011, beta = 0.754 95% CI (0.350–0.858); P value < 0.0001, respectively. Moreover, we found that fear from treating COVID-19 patients is associated with poor practice (beta = − 0.634 95% CI (− 0.843, − 0.12), P value = 0.016).

**Table 7:** Results of multiple linear regression on factors associated with dentist practice score toward Covid-19

	Unstandardized β	95%CI	P value
*Practice score*			
Knowledge score	0.319	(0.192, 0.445)	< 0.0001*
Designation (specialist vs general dentist practitioner)	0.754	(0.350, 0.858)	< 0.0001*
Completing training on COVID-19 (Yes vs No)	1.234	(1.283, 2.185)	0.011*
Afraid to treat a suspected or confirmed patient infected with COVID-19? (Yes vs No)	− 0.634	(− 0.843, − 0.12)	0.016*

*95%CI* 95% confidence Interval

^*^P value < 0.05 is considered significant

## Discussion

To the best of our knowledge, this is the first cross-sectional survey conducted to assess the knowledge and practice of dentists toward COVID-19 in Lebanon. Our results showed that the majority of the Lebanese dentists had good knowledge while approximately half of the respondents had poor practice regarding COVID-19. The main common information source was the World Health Organization. The study also highlighted the fear of getting infected while working during the current viral outbreak. Moreover, our results showed that specialist dentists who completed training on COVID-19 with a high level of knowledge had better practice.

The findings of the current survey demonstrated that the majority of dentists had good knowledge (91.3%). Our result is consistent with a multinational study (92.7%) conducted by Kamate et al. [13] and another study conducted by Saqlain et al. [14] in Pakistan (93.2%). We also found that 73.37% of dentists used official government websites such as the World Health Organization as the main source of information about COVID-19. This indicates that the COVID-19 updates posted online by official government health authorities had positive implications for improving dentist knowledge levels.

In the present study, nearly half of the respondents (58.7%) followed precautionary measures. The poor practice of our dentists was observed in terms of providing patients with alcoholic disinfectants and masks in the waiting rooms (66.8%). Besides, there was a lack of disinfecting surfaces and fomites every 2 h with the appropriate sterilizer (62.8%). These gaps lead to a further viral spread in the community. The Lebanese order of dentists should release the appropriate recommendations to all registered dentists during the crisis to make sure that they are well informed and aware of the appropriate practice. Moreover, dentists need to adhere to the practice guidelines in order to provide safe environment to themselves and their patients. For example the PPE may create an efficient block against most potential dangers of aerosols produced from the operative area, the protective glasses or face shield should as well as a higher level of respiratory protection should be considered by the oral surgeon and the dental team to confront the novel virus. After each patient’s visit, surfaces should disinfect inert surfaces using chemicals confirmed against COVID-19 and keep a dry atmosphere to mitigate the 2019-nCoV spread.

Our multiple linear regression showed that being knowledgeable dentist leads to better practice toward COVID-19. Findings also showed that completing a COVID-19 training is significantly reflected by good practices. The lack of educational training may be attributed to some barriers such as the inability of some facilities to conduct educational face-to-face sessions. Online courses should be the alternative solution, delivered by the Lebanese order of dentists to encourage the dentists to comply with the infection control guidelines. Supplementary measures should be taken by the Lebanese order of physicians who should put forward their recommendations for dental services during the COVID-19 crisis to ensure the quality of infection control and publish their measures on their website and send messages and emails to the Lebanese dentist to keep them updated for safe infection control practices when dental professionals return to seeing patients.

It was observed that the specialist dentists reported better practice compared to the general practitioner. Kamate et al. [13] reported in his study that good practice was associated with qualifications. The possible explanation might be that specialist dentists perform research that updates the dentist’s knowledge based on recent guidelines and evidence-based practice.

The current study found that the majority of dentists were afraid to get infected from patients (86.3%) or their colleagues (81.8%). The response is similar to a study conducted among dentists from various countries, where they reported their fear of getting infected while working during the current viral outbreak [15]. Moreover, we found that fear of treating COVID-19 patients is associated with poor practice. Hence, psychological interventions to improve dentists’ mental health and to enhance confidence in the dentists’ ability to treat patients and return to their practice during the COVID-19 epidemic are needed in Lebanon.

### Recommendations for dental routine practice in response to COVID-19 outbreak in Lebanon

There is a growing awareness of the importance of handwashing among dental practitioners. Epidemiological studies have shown that proper hand hygiene by washing hands with soap and water, and cleaning hands with 70–90% alcohol-based hand rubs (ABHR) was effective in controlling the spread of Severe Acute Respiratory Syndrom (SARS) [16, 17]. Dentists should wash their hands before the patient examination, before dental procedures, after touching the patient, after touching the surroundings and equipment without disinfection. All the team in the clinic (dentists and assistants) should receive training on personal protective equipment (PPE) and demonstrate competency with proper use (e.g., putting on and removing without self-contamination). PPE includes protective eyewear, masks, gloves, caps, face shields, and protective outwear. It is strongly recommended for all healthcare givers in the clinic. Respirators that offer a higher level of protection such as N95, European Standard Filtering Face Piece 2 (EU FFP2), or equivalent were recommended when performing aerosol-generating procedures (using high-speed and piece, air–water syringe, and ultrasonic scaler).

Evaluation of patients in the clinic by measuring the body temperature in the first place. A contact-free forehead thermometer is strongly recommended for the screening. Clinics should provide supplies such as masks, alcohol-based hand rub with 60–95% alcohol, tissues, and no-touch receptacles for disposal, at healthcare facility entrances, waiting rooms, and patient check-ins. Dental clinics should also be frequently cleaned and disinfected, including door handles, chairs, and desks.

The dental clinics should assess the patient’s dental condition by phone. a questionnaire should be used to screen patients with potential infection of COVID-19 before visiting the clinic. These questions should include the following: (1) Do you have a fever or experienced fever within the past 14 days? (2) Have you experienced a recent onset of respiratory problems, such as a cough or difficulty in breathing within the past 14 days? Separate appointments should be scheduled at least one hour apart between patients who should be placed in a ventilated waiting area.

Antimicrobial mouthwashes containing agents such as chlorhexidine (CHX) have shown effectiveness against various respiratory viruses [18]. Hence mouth rinse before each dental procedure can reduce the load of oral microorganisms [19]. Also, the use of the rubber dam provides an excellent barrier to the potential spread of infectious disease in the dental office. Its application in aerosol-generating procedures reduced the spread of microorganisms by 90% [20].

Like the rest of the educational activities, dentistry students should enroll in online-based education. This new shape of learning is helpful to avoid exposure risk and provides a safe environment without interrupting any academic requirements.

In general, dentists should be aware of the COVID-19 critical situation and should comply with the standard measures needed to improve the infection control strategies during this epidemic. Further recommendations for dental treatment can be found elsewhere in relevant documents [21, 22].

### Limitations

Our study had some limitations. Due to the lockdown, we adopted the snowball sampling strategy which was not based on a random selection of the sample, and the findings did not represent all the Lebanese dentists and therefore our findings cannot be generalized. Also, the cross-sectional nature of the study can only demonstrate association and not a cause-effect relationship. Despite the limitations identified, we believe that the study addresses a major health problem that challenges dentists in Lebanon.

## Conclusion

Dental professionals, by nature, are at high risk of exposure to infectious diseases. The emergence of COVID-19 has brought new challenges and responsibilities to dentists. Lebanese dentists revealed good knowledge regarding COVID-19. However, dentists had limited comprehension of the extra precautionary measures that protect the dental staff and patients from this virus. Our findings have important implications for the development of strategies suitable for improving the level of practice among dentists and enhance prevention programs. The implementation of special precautions can prevent disease spread from patients and serve as a guide for managing other respiratory diseases in the future.

## Supplementary information

Additional file 1 Study questionnaire.

## Data Availability

Data are available from the corresponding authors upon reasonable request.

## Abbreviations

COVID-19: Coronavirus Disease 2019
SARS-Cov-2: Severe acute respiratory syndrome coronavirus 2
WHO: World Health Organization
CDC: Centers of disease controlled and prevention
SD: Standard deviation
CI: Confidence interval
SPSS: Statistical Package for Social Sciences
MOPH: Ministry of Public Health
TV: Television
ABHR: Alcohol-based hand rubs
SARS: Severe Acute Respiratory Syndrome
PPE: Personal protective equipment
EU FFP2: European Standard Filtering Face Piece 2
CHX: Chlorhexidine
IRB: Institutional Review Board
ZHUMC: Al Zahraa Hospital University Medical Center
