# Supplementary material for: Assessment of knowledge and practice of dentists towards Coronavirus Disease (COVID-19): a cross-sectional survey from Lebanon
Source: BMC Oral Health. 2020 Oct 13;20:281. doi: 10.1186/s12903-020-01273-6 (PMC7552581; doi:10.1186/s12903-020-01273-6)
Supplement: Supplementary file 1 — Study questionnaire. [file 12903_2020_1273_MOESM1_ESM.docx]

**Questionnaire**

**Please respond to the following questions by placing a check mark (√) in the answer box that corresponds to your response and/or fill in the blank where indicated.**

1. Baseline Characteristics

| **Age** in years | **------------------------------------** |
| --- | --- |
| **Clinical Experience in years** | **------------------------------------** |
| **Gender** |  |
| Male |  |
| Female |  |
| **Marital status** |  |
| Married |  |
| Others |  |
| **Household** |  |
| No partner |  |
| Partner |  |
| **Designation** |  |
| General dentist practitioner |  |
| Specialist |  |
| **Completing training on COVID-19** |  |
| No |  |
| Yes |  |
| **Treating COVID-19 patients in special dental clinics** |  |
| No |  |
| Yes |  |

1. Knowledge Assessment.

| Items | True | False | Don’t know |
| --- | --- | --- | --- |
|  |  |  |  |
| K1. The incubation period of Coronavirus is 1–21days |  |  |  |
| K2. The main symptoms of Corona are fever >38°C, cough, sore throat, runny nose and shortness of breath |  |  |  |
| K3. Corona virus does not infect children |  |  |  |
| K4. Covid-19 can be prevented by administration of a vaccine |  |  |  |
| K5. Covid-19 is transmitted through direct contact with respiratory tract secretions |  |  |  |
| K6. Covid-19 can persist on surfaces for a few hours or up to several days, |  |  |  |
| K7. Covid-19 can be transmitted through eating undercooked meat/chicken |  |  |  |
| K8. The disease cannot be transmitted from asymptomatic patients |  |  |  |
| K9. The use of Personal protective equipment (including masks, gloves, gowns and goggles or face shields) is recommended to protect skin and mucosa from (potentially) infected blood or secretions |  |  |  |
| K10. Hand hygiene has been considered the most critical measure for reducing the risk of transmitting of Coronavirus to patients |  |  |  |
| K11. All surfaces contaminated by the patients with Covid-19 infection should be cleaned with diluted (5%) bleaching solution |  |  |  |
| K12. Dentists should take strict personal protection measures and avoid or minimize operations that can produce droplets or aerosols. |  |  |  |
| K13. PPE donning sequence: 1) gown 2) mask 3) gloves |  |  |  |
| K14. PPE removal sequence: 1) gloves 2) gown 3) mask |  |  |  |

1. Where do u get your information on Covid-19 from? Check all that apply

| World Health Organization |  |
| --- | --- |
| Ministry of Public Health |  |
| Television |  |
| Social media |  |
| Lebanese order of dentists |  |
| Centers for Disease Control and Prevention. |  |
| Others |  |

1. Assessment of dentist practice in the clinic after announcing the first positive corona case in Lebanon.

| Items | Always | Occasional | Never |
| --- | --- | --- | --- |
|  |  |  |  |
| P1. I clean my hands by using alcohol-based hand rub or soap and water |  |  |  |
| P2. I Clean and disinfect environmental surfaces |  |  |  |
| P3. I Wear the personal protective equipment such as dental goggle, mask, gloves, face shield, head cover and feet cover (dentist) |  |  |  |
| P4. I wear the personal protective equipment such as dental goggle, mask, gloves, face shield head cove and feet cover (assistant and team) |  |  |  |
| P5. I wash my hands before and after patient treatment |  |  |  |
| P6. Change gloves after each patient |  |  |  |
| P7. I Perform hand hygiene Before putting on gloves and again immediately after removing gloves. |  |  |  |
| P8. I Avoid busy clinic and I give separate appointments |  |  |  |
| P9.I provide patients with alcoholic disinfectants and masks in the waiting rooms |  |  |  |
| P10. I Disinfect all surfaces, chairs, and doors of the waiting room every 2 hours with Chlore solution or any type of sterilizer |  |  |  |
| P11. I Disinfect the patient’s chair and light between the patient and the other |  |  |  |

1. Assessing the fear of dentists toward COVID-19.

|  | Yes | No |
| --- | --- | --- |
| Are you afraid to treat a suspected or confirmed patient with COVID-19 in your clinic? |  |  |
| Are you afraid of getting infected with COVID-19 from a colleague? |  |  |
| Did your assistant express his/her desire to stop work due to fear of infection with the Coronavirus |  |  |
| Are you afraid of the impact of COVID-19 crisis on dentists’ livelihood |  |  |

**How would you rate the actions/policies are taken by your health care facility in fighting COVID -19**

- No appropriate actions were taken by the order of dentists regarding COVID-19
- The actions were insufficient
- The actions were acceptable/appropriate to combat COVID-19.
